# A Photography-based, Social Media Walking Intervention Targeting Autonomous Motivations for Physical Activity: Semistructured Interviews With Older Women

**DOI:** 10.2196/35511

**Published:** 2022-04-14

**Authors:** Michael C Robertson, Maria Chang Swartz, Ursela Christopherson, Jason R Bentley, Karen M Basen-Engquist, Debbe Thompson, Elena Volpi, Elizabeth J Lyons

**Affiliations:** 1 Department of Nutrition, Metabolism & Rehabilitation Sciences School of Health Professions University of Texas Medical Branch at Galveston Galveston, TX United States; 2 Department of Pediatrics-Research University of Texas MD Anderson Cancer Center Houston, TX United States; 3 Department of Clinical Health and Applied Sciences University of Houston – Clear Lake Houston, TX United States; 4 Department of Behavioral Science University of Texas MD Anderson Cancer Center Houston, TX United States; 5 US Department of Agriculture/Agricultural Research Service Children’s Nutrition Research Center Baylor College of Medicine Houston, TX United States; 6 Sealy Center on Aging University of Texas Medical Branch Galveston, TX United States

**Keywords:** physical activity, walking, exercise, fitness, social media, health, intervention, behavior, behavior mechanism, psychological theory, serious games, gamification, older women, older adults, behavior change, behavioral interventions, mobile phone, photography, patient perspective, patient attitude

## Abstract

**Background:**

Older adult women are at risk for negative health outcomes that engaging in sustained physical activity can help prevent. However, promoting long-term maintenance of physical activity in this population has proven to be a challenge. Increasing autonomous motivations (ie, intrinsic, integrated, and identified regulations) for physical activity may facilitate enduring behavior change. Digitally delivered games for health that take a celebratory technology approach, that is, using technology to create new ways to experience valued behaviors and express valued beliefs, may be a useful way to target autonomous motivations for physical activity. Formative research with the target population is needed to design compelling intervention content.

**Objective:**

The objective of this study is to investigate older adult women’s reactions to and thoughts about a photography-based, social media walking game targeting autonomous motivations for physical activity.

**Methods:**

During an individual semistructured interview, a moderator solicited feedback from 20 older adult women (age range 65-74 years) as part of formative research to develop a social media game featuring weekly walking challenges. The challenges were designed to target autonomous motivations for physical activity. Interviews were audio-recorded and transcribed verbatim. Two reviewers conducted thematic content analysis on interview transcripts.

**Results:**

We identified 3 overarching themes in qualitative data analysis. These reflected the playful experiences, value, and acceptability associated with the intervention challenges. Generally, participants understood what the challenges were asking them to do, proffered appropriate example responses, and indicated that the challenges would be enjoyable. Participants reported that the intervention content afforded many and varied playful experiences (eg, competition, discovery, exploration, expression, fellowship, humor, nurture, sensation). Further, participants indicated that the intervention increased their motivation for physical activity, occasioned meaningful shifts in perspective, increased their knowledge of various topics of interest, provided an opportunity to create valued connection with others, and provided health-related benefits. Participants suggested the intervention emphasize local history, nature, and cultural events.

**Conclusions:**

The photography-based, social media walking game with relatively simple game mechanics was well received and judged to be apt to bring about a wide variety of emotive experiences. A clear, geographically specific identity emerged as a key driver of interest for intervention content. Taking a celebratory technology approach holds promise for targeting autonomous motivations for physical activity in older adult women.

## Introduction

Older adult women are at risk for negative health outcomes, including chronic health conditions, cognitive decline, mental health challenges, fall-related injuries, decreased physical functioning, and decreased quality of life [1-8]. Physical activity may help protect against these negative health outcomes and is recommended for older adults by authoritative sources [9-12]. However, most older adult women do not engage in recommended levels of physical activity [13,14].

Behavioral interventions to increase physical activity in older adult women have been relatively successful in promoting physical activity in the short term [15]. However, to maximize the health-protective benefits of physical activity, long-term physical activity maintenance is necessary. Consistently achieving long-term behavior change in this population has proven to be an elusive target [16-18]. This may be attributable in part to the theoretical underpinnings of most physical activity interventions. Many interventions have been based on social cognitive theory or notions of self-regulation and have placed great emphasis on the importance of skill building [18]. Although this approach has proven useful for promoting behavior change *initiation*, to achieve changes in long-term *maintenance* of physical activity, behavioral interventions may need to additionally target disparate processes.

The Maintain Identify Transformation (IT) model posits that *centered identity transformation*, the process of integrating a behavior with one’s internal self-representations, may facilitate the activation of efficient cognitive processing and, in turn, facilitate enduring behavioral changes [19]. Conversely, it holds that behavior change approaches centered on executive functioning, including those based primarily on social cognitive theory and promoting active self-regulation, are unlikely to lead to long-term maintenance of behavior change. This is in part because executive functioning is relatively slow, error prone, and impaired by adverse physiological and psychological states [19]. Targeting motivational constructs may be 1 way to help elicit centered identity transformation and the activation of some of the more efficient cognitive processes needed to support robust, sustained changes in health-related behavior [19].

Self-determination theory (SDT) provides a nuanced understanding of motivation for physical activity [20,21]. It discriminates intrinsic motivation (ie, finding the behavior to be inherently enjoyable) from extrinsic motivation (ie, engaging in a behavior as a means to an end). Extrinsic motivations are further parsed conceptually according to the degree to which the motivation is external (eg, rewards/punishment) versus internal (eg, valuing the behavior’s outcomes or identifying as a person who engages in the behavior) in nature. Increasing one’s autonomous motivations, which include both intrinsic motivations as well internal forms of extrinsic motivation (ie, *integrated* and *identified* regulations, which reflect valuing and identifying with the behavior and its consequences), may facilitate centered identity transformation [19]. SDT suggests that integrated and identified regulations for health-related behaviors are modifiable constructs that may increase as one’s core psychological needs are satisfied (ie, *autonomy*, *relatedness*, and *competence*).

Digitally delivered interventions hold promise for physical activity promotion in older adults [22]. Most digitally delivered interventions for physical activity take a *corrective approach* to health promotion—they are typically centered on redressing problematic aspects of one’s behavior. Such interventions tend to use technology to promote self-regulation and increase external forms of motivation (eg, the provision of wearable devices to provide feedback, data visualization schemes to foster comparison of behavioral performance with goals, and the provision of reward for performance) [23]. Taking a *celebratory technology approach* instead, that is, using technology to create new ways to experience valued behaviors and express valued beliefs, may help frame personal informatics more toward promoting reflection [24], context [25], and storytelling [26]. This may be more conducive to increasing autonomous motivation for physical activity, facilitate centered identity transformation, and lead to more robust, long-lasting behavior change.

Games for health, which use game design elements to achieve nongame objectives, can be used to target SDT constructs in a celebratory manner [27-29]. Games for health oriented toward health promotion emphasize the importance of pairing enjoyable experiences with the promotion of health-related behaviors [30]. They are a natural fit for taking a celebratory technology approach (vs a corrective approach). Rather than attempting to make it *easier* to be physically active, this approach to physical activity promotion may be centered on making it more *interesting* to be active. This represents a noteworthy departure from contemporary health promotion frameworks, and formative research with priority populations is needed to design compelling game content. We previously used the Mechanics, Experiences, Change (MECHA) model, a step-by-step program-planning model for designing games for health, to develop Challenges for Healthy Aging: Leveraging Limits for Engaging Networked Game-based Exercise (CHALLENGE) [31]. CHALLENGE is an autonomy-focused game for health that implements weekly walking challenges using social media. Despite systematic program design, making physical activity more interesting is an inherently challenging endeavor, and collaboration with the target population is needed [32]. It is likely that individual preferences and interests contribute to what people find interesting, so in-depth formative work is critical to the success of such a project. Thus, the purpose of this study is to interview older adult women to investigate the acceptability of this unique approach to physical activity promotion and how we might improve upon CHALLENGE.

## Methods

### Study Design and Recruitment

This study was a qualitative, formative study conducted prior to a randomized controlled trial. We recruited 20 participants to expand and refine CHALLENGE (presented later). Eligibility criteria included that participants be female adults between 65 and 85 years of age who were able to read and understand English. We excluded the “oldest old” population due to differences between this population and younger-old populations who tend to have fewer functional limitations [33] and because this younger-old population has demonstrated general acceptance and enjoyment of mobile and wearable devices [34,35], games [36,37], and mobile health (mHealth) interventions [38,39]. During the COVID-19 pandemic, we altered study protocols to allow for online interviews. These changes further necessitated that participants have access to teleconference capability from home (eg, Skype, FaceTime, or Zoom). We recruited participants via in-person recruitment at University of Texas Medical Branch (UTMB) gerontology and primary care clinics, online recruitment using the UTMB’s newsletters, and flyers and brochures available at locations frequented by older adults. Originally, 20 participants were recruited for this study. However, 1 (5%) participant’s audio data failed during her videoconference and thus could not be used. Due to this issue, the research team recruited an additional participant to reach the target sample size.

### Ethics Approval

This project was approved by the UTMB at Galveston’s Institutional Review Board (protocol no. 19-0158). Verbal informed consent was obtained from all participants prior to participation in this study.

### Intervention

We developed CHALLENGE using the MECHA model [31]. The details of this game for health physical activity intervention are presented elsewhere [31]. Briefly, CHALLENGE featured weekly walking challenges to be delivered via a private Facebook group. Rather than promoting performance-based achievements (eg, obtaining a particular step count), the weekly challenges were designed to elicit playful experiences (PLEXs) and target SDT psychological needs (ie, autonomy, competence, and relatedness) and autonomous motivations (ie, intrinsic, integrated, and identified regulations) to encourage walking behaviors [40,41]. For example, 1 weekly challenge invited participants to present a photograph of an object that might serve as a symbol of their community.

### In-Depth Interviews

From January to December 2020, we conducted an in-depth, semistructured interview with each participant to investigate perceptions of the feasibility and acceptability of the weekly walking challenges. We conducted interviews in 2 rounds, which were separated by study staff revising CHALLENGE content based on participant feedback. The first round of interviews was conducted in person. Due to closures to research in March 2020 associated with the COVID-19 pandemic, the second round of interviews was conducted via videoconference. A trained research assistant took a modified cognitive interview approach, in which she presented all challenges to the participants and asked them to read each challenge aloud and talk through their impressions of the challenge. An example challenge is

Party Crasher. Find an animal party. Go on a walk and look for a group of birds, squirrels, cat, dogs, or any animal socializing around your neighborhood. Snap a photo and share it. Crash the biggest animal party you can find!

The research assistant responded with probing questions concerning (1) what the participant thought the challenge writer meant, (2) what they believed the challenge writer wanted them to do, (3) what photos they might take to fulfill the challenge, (4) what was the likelihood that they would accept the challenge, (5) what was the likelihood they would enjoy the challenge, and (6) what was the likelihood the challenge would lead to discussion. Interviews took about 2 hours to complete. Participants were compensated with a US $25 gift card. All interviews were digitally recorded and professionally transcribed verbatim.

### Qualitative Data Analysis

We imported interview transcripts from both rounds into the qualitative data analysis management program Nvivo version 12 (QSR International Pty Ltd). Two independent reviewers (authors MCR and EJL) conducted thematic content analysis on all interview transcripts using a hybrid approach to assign deductive codes and inductive codes to discrete points made by study participants [42]. The coders started with deductive codes, which reflected the conceptual model of the intervention. These codes reflected the PLEXs targeted by CHALLENGE and were derived from the PLEX framework (eg, *exploration*; Table 1) [40,41]. Inductive codes were used to capture other important or related information in the transcripts that were judged to be important in relation to the research questions (eg, *real-life benefits* [of the intervention] or *impacts of where you live* [on completing challenges]). After coding was complete, the inductive codes were examined and grouped into higher-order categories or themes (eg, impacts of geographic location, suggestions for improvement). The coders maintained a codebook and organized codes into recurring themes and subthemes. They regularly met to reach consensus regarding discrepancies in coding via discussion, identify recurring themes and subthemes, establish the overarching structure of themes and subthemes, and identify illustrative quotations.

**Table 1:** Definition of PLEX^a^ targeted by challenges.

PLEX	Definition [41]
Captivation	Forgetting one’s surroundings and the passing of time, absorption
Challenge	Testing abilities in a demanding task, developing and testing skills, learning new things
Competition	Contesting with oneself or an opponent, trying to achieve a defined goal
Completion	Finishing a major task, experiencing closure related to a tension, mastery experience, finishing a level or a game
Control	Dominating, commanding, and regulating; a feeling of being powerful
Cruelty	Causing mental or physical pain, feeling malicious or manipulative, bullying or destruction
Discovery	Finding something new or unknown, uncovering something previously hidden, surprise
Exploration	Investigating an object or situation, curiosity and a thirst for knowledge
Expression	Manifesting oneself creatively; designing, constructing, personalizing
Fellowship	Friendship, communality, or intimacy; sharing experiences with others
Humor	Fun, joy, amusement, jokes, gags
Nurture	Taking care of oneself or others, tutoring and helping others in the game
Relaxation	Relief from bodily or mental work, “unwinding” while playing the game
Sensation	Excitement by stimulating senses, pleasure related to senses
Subversion	Breaking social rules and norms or seeing others do so, twisting meaning
Suffering	Experience of loss, frustration, and anger during play
Thrill	Excitement derived from risk and danger, suspense and excitement

^a^PLEX: playful experience.

## Results

### Participant Characteristics

Of the 20 participants constituting the analytical sample, 15 (75%) identified as non-Hispanic White, 3 (15%) identified as Hispanic, and 2 (10%) identified as Black. The average age was 70.1 years (SD 2.6, range 65-74).

### Thematic Content Analysis

Two rounds of data collection were performed. The first round of interviews included 9 (45%) of 20 participants. Although we originally planned to update the challenges after the 10th participant, closures to research during March 2020 led to a natural stopping point after the 9th participant. We revised the challenges after the first 9 interviews based on participant feedback. We then proceeded to conduct interviews with 11 (55%) additional participants and the revised challenges. The average age and racial/ethnic makeup of the participants were nearly identical in rounds 1 and 2. We identified 3 overarching themes in our qualitative data analysis. These were (1) PLEXs, (2) value derived from the challenges, and (3) acceptability of the challenges.

#### Playful Experiences

All but 1 of the PLEXs outlined in the PLEX framework were mentioned by participants (*control* was never mentioned by the participants). Table 2 presents illustrative quotes for each of the PLEXs observed. The most commonly occurring PLEXs were competition, discovery, exploration, expression, fellowship, humor, nurture, and sensation.

*Competition* was the most frequently occurring code. However, discussion of interindividual competition was rare. Discussions of competition were largely centered on an individual-level, low-stake pursuit of trying to achieve a defined goal (eg, finding something specific while walking). Overlapping with experiences of competition, participants commonly articulated experiences of *exploration* and *discovery*. This cluster of codes often reflected a straightforward response to the intervention prompts, typically centered on walking in novel environments with openness and curiosity or physically finding specific items participants felt would constitute suitable responses to the challenges. However, notions of exploration and discovery also sometimes included more abstract discussion of encountering emotive circumstances or discovering new perspectives toward familiar objects. For example, 1 (5%) participant described her pleasant experiences walking through antique stores.

I love to walk through antique stores, and it does make me walk. And I will go to a historic district that has a lot of antique stores, and I don’t even have to buy anything anymore, because I’ve got enough junk, but it’s so warming to walk through it.

P11, 69 years old

Participants’ experience of *expression* often reflected their perceptions of their own artistic inclinations and the implications of this for their enjoyment (or lack thereof) of certain challenges. Much discussion linked to experiences of expression was centered on engaging in the creative process or communicating one’s identity and values to others in the social group. Numerous participants, for example, expressed pride in their Texan identity. One participant stated,

I’ve got, for instance, on the front of my house, I’ve got a decoration that has the state of Texas shape on it. I could take a photo of that and explain why I bought that.

P17, 74 years old

**Table 2:** Illustrative quotes for each PLEX^a^.

PLEX	Illustrative quote
Captivation	“You have to clear your mind and just put aside all your preconceived notions of what you’ve seen and done before, and then look at it like somebody that really never has been there.” [P15, 65 years old], where “P” stands for “participant.”
Challenge	“It would be interesting because my tree knowledge is basic. I did not enjoy my forestry class, back in the sixties, but I keep wanting to revitalize some of my tree knowledge, so I would find this one interesting. I'd probably do it more than twice.” [P12, 73 years old]
Competition	“I think this one’s more about individual competition. How many did you find and tell us. It’s a count thing, so if I don’t find them but I went out and walked, am I a loser?” [P19, 65 years old]
Completion	“It’s something that I would like to do because I would want to get them all and say, ‘See, I got them all.’ That’s just me.” [P6, 68 years old]
Cruelty	“Go to the yacht basin and watch the tourists try to launch their boats. That’s always funny.” [P15, 65 years old]
Discovery	“Find something that piques our curiosity. And there’s a lot of things. Certain buildings. What was the history of that building? Who lives there? Or just different things. I think it’d be interesting.” [P10, 70 years old]
Exploration	“To take different routes…to walk in a different way…to go in different areas to walk. Don’t walk in the same way all the time. Don’t be a creature of habit. Take a chance. Do something different.” [P5, 72 years old]
Expression	“I think that’s such a personal thing, what people appreciate. One person can say, ‘I’m thankful for the house that I live in.’ Another person could say, ‘I’m thankful for living in Houston or this environment.’ You can take pictures of your family…anything like that. It would really depend on the person, I think.” [P21, 71 years old]
Fellowship	“…it would initiate conversation. And I need that as much as I do physical activity.” [P11, 69 years old]
Humor	“I would just be taking a walk and take a picture of something that made me smile or chuckle, which could be…sometimes, I see a beautiful sunflower, and it just brightens and lightens and [makes me] smile.” [P15, 65 years old]
Mindfulness^b^	“Oh, I think it’s instead of just walking swiftly through to get to your designation, it’s like you notice that some of these streets have bricks on the street to walk on or some of them are higher up where you have to step down.” [P4, 72 years old]
Nurture	“And in the spirit of the program, everybody should be encouraging everybody to go see this or try it or do this or do that.” [P9, 68 years old]
Relaxation	“So, I think whatever you do should be relaxing and nurturing and calming. And it could be just watching waves come in and out. It could be walking through a forest or like a nature park somewhere here in Greater Houston.” [P16, 71 years old]
Sensation	“I’m thinking of the rose garden out at the UTMB^c^, which is a nice little, short walk, and the discussion around that could range from, yeah, some of them have no smell at all, but they’re gorgeous, and the really old tea flowers, the tea roses, have [a] really nice scent, so there could be discussion about size, color, and scent.” [P12, 73 years old]
Subversion	“My husband found a picture on the internet of a cloud that was shooting somebody, the bird. Now, if you don’t think I wouldn’t put that picture on, you’re sadly mistaken, because I would.” [P5, 72 years old]
Suffering	“I could end up getting stressed out after 10 or 20 of these if trying to find things that I just don’t usually see.” [P16, 71 years old]
Thrill	“We’ve got action scenes. We’ve got—the police are always out on the seawall, so there’s always a cop-type chase.” [P10, 70 years old]

^a^PLEX: playful experience.

^b^This experience was not originally featured in the PLEX framework but emerged as an important experience in this study.

^c^UTMB: University of Texas Medical Branch.

Discussions of *fellowship* included sharing or bonding with other participants in the group and having real-world interactions with others (eg, physically meeting with other participants, involving existing walking partners or acquaintances in the walking game). In this context, participants also commonly discussed experiences of *nurture*. These discussions were characterized by prosocial intentions to provide emotional nutriment (eg, positive emotional supportive) or instrumental support (eg, hurricane preparedness tips) to others. Participants also talked about intraindividual experiences of nurture. Such comments were generally centered on engaging in personal reflection or experiencing catharsis that could include feelings of appreciation or nostalgia.

Experiences of *suffering* were relatively rare and usually in reference to participant burden or uncomfortable internal sensations (both physical and emotional, such as reflecting on painful memories of powerful storms). Experiences of *subversion* typically reflected a breach of social norms and encountering potentially uncomfortable or potentially dangerous consequences (eg, reproach from individuals after photographing their likeness or identifying information, such as license plates). Although it is not outlined in the PLEX framework, we added *mindfulness* as a PLEX evoked by the challenges that was related to captivation conceptually but was ultimately judged to be distinct. Rather than forgetting the passing of time and one’s surroundings due to absorption in something else, mindfulness involved captivation by and absorption in one’s surroundings and the present moment. This code occurred frequently, and such comments typically reflected participants making efforts to purposively focus their attention on the present moment or become more aware of their environment while engaging in the walking challenges.

#### Value Derived From the Challenges

The second overarching theme we identified was centered on the value participants derived from the challenges. In total, 5 subthemes were reflective of this overarching theme. These were (1) increasing motivation for physical activity, (2) fostering a shift in perspective, (3) increasing knowledge, (4) making meaningful connections with others, and (5) providing health-related benefits.

Participants indicated that by pairing novel, game-like objectives to their walks, the challenges would imbue walks with a sense of directed purpose that could be motivating. Once participant said,

To look for things to make it more interesting to walk, because sometimes I think people get bored with just walking and them not having a mission or a purpose.

P5, 72 years old

Participants added that the other valued aspects of the challenges (ie, bringing about a shift in perspective, increasing knowledge, making meaningful connections with others, and providing health-related benefits) could further increase motivation for walking. For example, participants indicated that the *shift in perspective* brought about by some of the challenges could improve their autonomous motivation for walking behaviors. One participant stated,

And I always see new sunsets or—new sunsets or sunrises—so those are always new beginnings and new renewals. I mean, it seems like if I’m looking at new things or renewals or new beginnings, I would be more involved in my walk. I would have a purpose with my walk.

P10, 70 years old

Participants valued many aspects of *knowledge* and learning that the CHALLENGE study intervention featured. This included learning about natural phenomena (eg, types of cloud formations, local flora and fauna). One participant shared,

Well, if there’s a list of places to go for bird activity, I’d love to find that because my daughter-in-law is a big bird fan, and so she probably doesn’t know some places that you’ve got on your list. But that would be interesting because that would kind of force you out to go to a park or a sanctuary and look for the birds.

P19, 65 years old

Participants were also particularly interested in learning about the local attractions that the challenges celebrated (eg, Mardi Gras, Featherfest, Galveston Art Walk, Harvest Moon Regatta boat race, Dickens on the Strand festival), and frequently expressed interest in learning about or visiting specific locations related to the challenges (eg, the historic tall ship *Elissa*, the Seawall, the Strand). Another element of the challenges that participants valued learning about was South Texas history and culture. Many participants were interested in learning about Texas history (eg, historic buildings in Galveston, historic hurricanes) and lore (eg, purportedly haunted locations). These challenges stoked expression of some participants’ Texas identity. One participant stated,

I’m from Texas and I love Texas symbols, so I probably would be really interested to see what kinds of things are out there…and a lot of people are interested in the history of our state.

P3, 72 years old

Participants also valued the potential that engaging with game content may have for facilitating *connection* *with others*. First, participants indicated that the pursuit of completing game challenges may stimulate interesting discussion and knowledge sharing with friends and family members (eg, sharing interesting knowledge gleaned from the challenges). One participant indicated that the challenges would lead to “a lot of discussions. When I look back in history and think of the history and share it with my grandchildren” [P11, 69 years old]. Second, participants talked about including friends and family members in gameplay directly (eg, walking with family members and recruiting them to help craft responses to challenges). Third, participants talked about the prospect of interacting with members of the community during gameplay (eg, asking strangers for permission to photograph their property). Finally, participants valued potentially connecting with other participants in the Facebook group. Some participants were particularly enthusiastic about the prospect of contributing to an uplifting and supportive environment.

Participants made comments indicating that they valued the health-related benefits of increased walking. However, these comments were relatively infrequent. Such comments were relatively general in nature (eg, physical activity facilitating longevity) and did not pertain to specific health-related benefits. For example, 1 participant indicated that she appreciated that the intervention would encourage her to “take pictures of moving things so that you get off your butt and on your feet, because being sedentary can only shorten your life” [P5, 72 years old].

#### Acceptability of the Challenges

Responses indicated that most of the challenges and the proposed intervention delivery modalities (eg, Facebook) were acceptable. Of the 63 challenges included in the first rounds of interviews, we kept 48 (76%) and removed 15 (24%) based on participant feedback. In the second round, we included 59 challenges in the interview guide, 14 (24%) of which were new (some challenges were combined based on the advice of participants from the first round). Generally, participants understood what the challenges were asking them to do, proffered appropriate example responses, and indicated that the challenges would be doable and enjoyable. Participants appreciated challenges that they felt were both interesting and convenient in the context of their regular routines. Table 3 presents some of the challenges that garnered the most enthusiasm. Participants also appreciated ancillary material provided by CHALLENGE, which included checklists (eg, of local birds, types of clouds), badges (ie, graphic images associated with each challenge awarded to respondents), and props given at the beginning of the study to facilitate responding to certain challenges (eg, silly sunglasses to help conceal identity in selfies).

Participants indicated that some of the challenges presented were not acceptable. Examples of challenges with low acceptability are presented in Table 4. These challenges were typically rejected because they were on a topic the participants did not like (eg, tattoos), required behaviors they did not want to do (eg, pick up garbage), or were felt to be confusing or too abstract (eg, coming up with puppy names). We also removed some challenges that were highly rated in a few cases where participants brought up safety concerns (eg, participants were worried that rocks featured in 1 challenge could be used by others to damage property and that taking photos of out-of-state license plates could provoke anger).

**Table 3:** Examples of challenges with high acceptability (based on most comments).

Challenge topic (description)	Illustrative quote
Arborist (invited participants to share photos of various trees presented in an ancillary checklist)	“Well, just paying attention to the trees and looking at them and figuring out, well, what type of tree is it? I think this would be really interesting. I would enjoy doing this.” [P17, 74 years old]
Change perspective (invited participants to view their environment from a different point of view)	“I think there’s this interesting fountain thing on the neighbor’s house entrance, and so you could see taking different times of day photos of something like that. That would be interesting, and it would force you to be out at different times.” [P19, 65 years old]
Detour-er (invited participants to share photos and take a different route from their normal routine)	“I’d take pictures of things I’d never seen before or didn’t know were there.” [P3, 72 years old]
Lone Star Bike Rally (referenced a Galveston Island tradition; invited participants to take pictures of interesting vehicles)	“This I could do. I have thousands of pictures I’ve taken over the years. We love that event. My daddy rode motorcycles. I was raised on them, so all my kids ride.” [P8, 71 years old]
Safari at home (invited participants share pictures of animals observed in their environment)	“Yeah, I think that would be interesting because it gives you a different way to look at your environment.” [P21, 71 years old]

**Table 4:** Examples of challenges with low acceptability (based on most comments).

Challenge topic	Illustrative quote
First week of winter (invited participants to share photos reflecting the coming of wintertime)	“But as far as our area, we’d be lucky if it’s even cold, you know.” [P8, 71 years old]
Neat freak (invited participants to share photos of picking up trash to beautify their environment)	“And would it generate a discussion? Just people like me griping about it.” [P6, 68 years old]
Punny ideas (invited participants to share photos reflecting plays on words)	“I might have to look up something on the web or something. I’m not very creative like that.” [P7, 71 years old]
Rebel rouser (invited participants to share photos of things that would make a good tattoo)	“I do not believe in tattoos. That would not be a good one for me. My grandson has tattoos all over his body. I just don’t think tattoos are cool. I don’t think you’re supposed to mark your body up like that.” [P5, 72 years old]
Terra feeling (invited participants to share photos of different terrains they have walked on)	“I don’t walk on anything that’s not paved. I don’t like to walk on grass, because there might be holes in it. I’m very careful where I walk.” [P3, 72 years old]

Nearly every participant suggested changes to challenges, and some participants offered suggestions for challenges that we adopted and subsequently proved to be popular; challenges dedicated to hurricane preparation, the Houston rodeo, and holiday traditions were suggested by participants and included in the final set of challenges. Further, participants provided numerous suggestions for revising presented challenges. Suggested revisions included changes in diction to increase clarity of the challenges and taking measures to provide participants with sufficient guidance for how to respond to challenges. Some challenges were open ended and abstract, and many of participants’ suggestions were centered on narrowing the scope of the challenges. Participants also recommended providing example responses following each challenge to facilitate better understanding of exactly how participants might respond. One participant stated,

I think having some of those colored examples and pictures for each of these challenges might actually [be] a good stimulant for us 65-and-older people who are not as creative.

P19, 65 years old

Thus, in the final intervention, we provide detailed example messages for all challenges. Somewhat related to this, some participants also proposed suggestions for minimizing participant burden. One participant stated,

Remember, older people, when you give them a whole lot of tasks to do, they get real anxious. So, I wouldn't give them more than 4 or 5.

P10, 70 years old

Finally, participants made suggestions for increasing the relevance and accessibility of the challenges. In most cases, these suggestions pertained to geographic location. The 20 participants in this sample lived in various areas of Southeast Texas and had different experiences of their surroundings. Participants emphasized the important role that one’s immediate environment would have in influencing their experience of the walking challenges. Some challenge content was centered on attractions related to a specific region (eg, Galveston Island). Although participants living within this area were often particularly excited about some of these challenges, some who lived outside of this specific area were less enthusiastic. One participant stated,

Again, if there were really historical markers in the Clear Lake area, yes. There’s so much Galveston-specific things here, and Galveston offers a lot. I don’t know how Clear Lake compares to it with something like this.

P17, 74 years old

Participants mentioned specific locations as places they would go to complete challenges. Most mentioned were Galveston Island proper and local landmark attractions, including the Seawall, the Strand downtown district, the beach, and the tall ship *Elissa*. Participants also commonly commented on how the specific details of their home environment (even within Galveston Island) would have a bearing on their responses to challenges. For example, 1 participant said,

Well, I’m going to tell you, that’s going to be hard, there again, because in my neighborhood, there’s nothing like that. I’ve seen it in Galveston where the stumps [have been fashioned] into beautiful pieces of art, but I don’t think I could do that here where I walk. This is where I limit myself. I feel safe walking here, and I don’t plan on walking anywhere else, you know?

P14, 69 years old

## Discussion

### Principal Findings

In this study, we conducted in-depth interviews with older adult women to investigate the acceptability of CHALLENGE, a digitally delivered game for health centered on the use of celebratory technology to target autonomous motivations for physical activity. We identified 3 overarching themes. These pertained to the PLEXs afforded by the behavioral intervention, the value participants derived from the weekly challenges, and the acceptability of the challenges. Overall, the challenges engendered many and varied PLEXs and participants valued the autonomy-supportive aspects of the behavioral intervention. Participants were particularly interested in challenges that targeted local history, nature, and cultural events, and encouraged us to emphasize these points in the final intervention. Participants provided useful, specific recommendations for adding, removing, and changing challenges that might facilitate targeting autonomous motivations for physical activity.

Our findings are concordant with other studies that have suggested that digitally delivered interventions may be useful for targeting autonomous motivations for health-related behaviors. A study conducted by Chen et al [43] found that a social photography intervention that had participants take daily photos that they believed would make themselves or others happy led to positive outcomes, including feelings of connection and reflection. A social media smoking cessation study found that interactive cocreation of health promotion materials improved information seeking and subjective norms as compared to simply viewing materials [44]. Finally, another trial investigated the effects of social media–delivered daily eating challenges that were either nutritionally prescriptive or nonnutritionally prescriptive (ie, less corrective, more celebratory in nature) [45]. The study found that the nonnutritionally prescriptive challenges were feasible, engaging, and associated with changes in learning about food and diet. Further, assignment to the nonnutritionally prescriptive arm was associated with greater increases in mindful eating than assignment to the nutritionally prescriptive arm. These findings support the conclusions of this study that a celebratory technology approach may hold promise for targeting autonomous motivations for health-related behaviors. More research is needed to better specify and evaluate corresponding mechanisms of successful, long-term behavior change.

PLEXs are important determinants of long-term adherence to digitally delivered physical activity interventions [46]. The findings of this study extend the previous literature by investigating the nature of the PLEXs afforded by a physical activity–promoting game for older adults. Participants indicated that intervention challenges elicited many and varied PLEXs. We initially developed CHALLENGE using the MECHA model [31]. The MECHA model was derived from the Behavior Change Wheel [47,48], and its use ensured specific linkages between game design elements and underlying SDT constructs. In the context of this study, it was particularly useful to have integrated the PLEX framework [40] in the design of CHALLENGE. This framework helped guide our qualitative data analysis to yield a more nuanced understanding of the emotive outcomes of the intervention. Games for health can lead to increases in older adults’ physical activity levels and mental and social well-being [49,50], and it may be that the emotive, PLEXs that they engender may mediate their outcomes. The PLEX framework has a quantitative measure that operationalizes its PLEXs and may be used to test such hypotheses [41]. In the randomized controlled study to follow this study, we aim to assess to what degree CHALLENGE elicits the desired experiences and whether this is associated with psychosocial and behavioral outcomes.

The previous literature suggests that it is advantageous to tailor physical activity intervention content to ensure its relevance to the target population [51]. Most individually tailored digital physical activity interventions are tailored to participants’ physical activity performance or stage of change [52]; however, tailoring to individuals’ cultural identities is also critically important [53,54]. Results of this study highlight the importance of tailoring intervention content to the target population’s identities and values. The challenges featured in this study were specific to Southeast Texas from the outset and became more so with refinement following participant feedback. Although the challenges could be completed anywhere, the intervention subject matter was often based on local history, nature, and cultural events. Throughout this study, participants suggested the intervention be expanded to include challenges based on specific local topics, such as historic hurricanes, specific locations, and local festivals. It is not clear to what degree such a specific focus would be necessary for similar interventions in other contexts, but a clear, localized identity emerged as a key driver of interest for this study sample. Although tailoring intervention content in this way may be particularly useful for targeting autonomous motivations, resulting interventions may not be as readily disseminated to other populations and contexts. Extensive qualitative research is likely necessary to create resonant behavioral interventions. A sense of identity as Texan was strong in our sample, but such a geographically oriented identity may not be salient for other populations. Nonetheless, it may be that the platform technologies and general intervention framework featured in this study can help facilitate efficient program adaptation. Content could be adapted for groups that share a common interest rather than geographically limited identity (eg, art, gardening, travel). Geographically limited challenges could also be themed to appeal to individuals who are interested in learning about the culture, history, and language of specific places where they do not necessarily live.

### Limitations

This study has several limitations. First, the generalizability of this study is limited by our convenience sampling recruitment method. Participants in our sample mostly identified as non-Hispanic White and may have been particularly receptive to behavior change as they opted to participate in research. Although in-depth, individual interviews are ideal for gathering particularly rich and extensive qualitative data, our study was further limited by a small sample size and the fact that we only interviewed participants on 1 occasion. It is possible that participant sentiment on the topics discussed may shift over time. Further, this study occurred in the context of the uncertainty and sweeping stay-at-home orders associated with the COVID-19 pandemic. These circumstances impacted individuals’ attitudes and propensity to participate in research [55]. Related, please note that personal safety was a major theme related to subversion that we identified in this study, but this topic was not covered here, because we judged the nuance involved to warrant more detailed discussion; these findings are to be presented in an upcoming report.

### Conclusion

In conclusion, in this study, we investigated older adult women’s reactions to a social media game designed to target autonomous motivations for physical activity. We identified 3 overarching themes, which reflected the experiences, value, and acceptability afforded by the intervention. Participants enumerated many and varied PLEXs associated with the intervention content and suggested that centering the behavioral intervention on a clear, localized identity would facilitate targeting integrated and identified forms of motivation for engaging in physical activity. This formative work may facilitate taking a celebratory technology approach to targeting autonomous motivations for physical activity in older adult women. Further research is needed to evaluate to what degree findings hold using different methods of assessment and in other populations.

## Abbreviations

CHALLENGE: Challenges for Healthy Aging: Leveraging Limits for Engaging Networked Game-based Exercise
MECHA: Mechanics, Experiences, Change
PLEX: playful experience
SDT: self-determination theory
UTMB: University of Texas Medical Branch

## Data Availability

The data that support the findings of this study are available from the corresponding author, EJL, upon reasonable request.
